# Vision transformer and explainable transfer learning models for auto detection of kidney cyst, stone and tumor from CT-radiography

**DOI:** 10.1038/s41598-022-15634-4

**Published:** 2022-07-06

**Authors:** Md Nazmul Islam, Mehedi Hasan, Md. Kabir Hossain, Md. Golam Rabiul Alam, Md Zia Uddin, Ahmet Soylu

**Affiliations:** 1 Department of Computer Science and Engineering, BRAC University, Dhaka, Bangladesh; 2 Radiology & Imaging Technology, Bangladesh University of Health Sciences, Dhaka, Bangladesh; 3 Department of Nephrology, Bangabandhu Sheikh Mujib Medical University, Dhaka, Bangladesh; 4 Software and Service Innovation, SINTEF Digital, Oslo, Norway; 5 Department of Computer Science, Norwegian University of Science and Technology, Gjøvik, Norway

**Keywords:** Biomedical engineering, Health care, Computed tomography

## Abstract

Renal failure, a public health concern, and the scarcity of nephrologists around the globe have necessitated the development of an AI-based system to auto-diagnose kidney diseases. This research deals with the three major renal diseases categories: kidney stones, cysts, and tumors, and gathered and annotated a total of 12,446 CT whole abdomen and urogram images in order to construct an AI-based kidney diseases diagnostic system and contribute to the AI community’s research scope e.g., modeling digital-twin of renal functions. The collected images were exposed to exploratory data analysis, which revealed that the images from all of the classes had the same type of mean color distribution. Furthermore, six machine learning models were built, three of which are based on the state-of-the-art variants of the Vision transformers EANet, CCT, and Swin transformers, while the other three are based on well-known deep learning models Resnet, VGG16, and Inception v3, which were adjusted in the last layers. While the VGG16 and CCT models performed admirably, the swin transformer outperformed all of them in terms of accuracy, with an accuracy of 99.30 percent. The F1 score and precision and recall comparison reveal that the Swin transformer outperforms all other models and that it is the quickest to train. The study also revealed the blackbox of the VGG16, Resnet50, and Inception models, demonstrating that VGG16 is superior than Resnet50 and Inceptionv3 in terms of monitoring the necessary anatomy abnormalities. We believe that the superior accuracy of our Swin transformer-based model and the VGG16-based model can both be useful in diagnosing kidney tumors, cysts, and stones.

## Introduction

Kidney disease is a public health concern since the disease is spreading despite current control attempts^1^. Chronic kidney disease affects more than 10% of the world population^2^, and it was ranked 16th among the leading causes of death in 2016 and is expected to jump to 5th by 2040^3^. Among the other kidney diseases, cyst formation, nephrolithiasis (kidney stone), and renal cell carcinoma (kidney tumor) are the most frequent kidney illnesses that impede kidney function. A kidney cyst is a fluid-filled pocket that forms on the surface of the kidney and is enclosed by a thin wall. Within the kidneys, one or more cysts may develop with water density: From 0 to 20 Hounsfield units^4–6^. Kidney stone disease is characterized by the formation of crystal concretions within the kidneys, which affects about 12% of the world population^7^. Renal cell carcinoma (RCC), often known as kidney tumor, is one of the ten most prevalent cancers in the world^8^.

X-ray, computed tomography (CT), B-ultrasound machines (US), and MRI (magnetic resonance imaging) machines are often used in conjunction with pathology tests to diagnose kidney diseases. The CT machine scans the desired part of the human anatomy with X-ray beams to obtain a cross-sectional image which provides three-dimensional information about the desired anatomy^9^. CT scans in kidney examinations are ideal for study because they provide three-dimensional information and slice-by-slice images. If kidney abnormalities such as cysts, stones, and tumors are not detected and treated early, they might lead to renal failure^10^. For this reason, early diagnosis of renal disorders like kidney cysts, stones, and tumors appears to be an important step in preventing kidney failure^11^.

On the other hand, the number of nephrologists and radiologist is very limited. In South Asia, there is barely one nephrologist per million people, where in Europe there are 25.3 nephrologists per million people^12^.

Considering the sufferings of the population due to kidney diseases, the shortage of nephrologists and radiologists around the globe, and the advancement of deep learning research in vision tasks, it has become imperative to build an AI (artificial intelligence) model to detect kidney radiological findings easily to assist doctors, and reduce the sufferings of people. A few studies have been published in recent years in this domain. However, the publicly available data set is scarce. In addition, most past studies have utilized traditional machine learning algorithms to classify single classes of disease only; either cysts, or either tumors, or either stones. Some studies utilised ultrasound (US) images.

In this work, we created and annotated the “CT KIDNEY DATASET: Normal-Cyst-Tumor and Stone” dataset^13^, implemented a total of six models, and evaluated each of them to come to the conclusion which model is best suited to use in realtime. The proposed auto-detection model for the diagnosis of kidney diseases will also help to build a digital twin of renal function at the pathology level, such as tumor growth. No study that we are aware of has done an analysis based on a transformer model with renal cyst, tumor and stone auto detection. The following are the major contributions of this work:

- A dataset namely “CT KIDNEY DATASET: Normal-Cyst-Tumor and Stone” is collected and annotated with 12,446 images utilizing the whole abdomen and the eurogram protocol.
- Three CNN-based deep learning models (i.e., VGG16, Resnet50, and Inception v3) using transfer learning approach are applied to detect kidney abnormalities and presented a thorough performance study, including explanation of the black-box of the suggested models using gradient weighted class activation mapping (i.e., GradCam).
- Three recent state-of-the-art Vision transformer variants (i.e., EANet, CCT, and Swin transformers) are applied on the CT kidney dataset and the performances of the models are presented using the confusion matrix, accuracy, sensitivity, specificity, and F1 score.

The rest of the paper is organized in the following manner. Section II provides background and details on utilizing deep learning to identify kidney abnormalities. The methodology for this letter is discussed in Section III, which includes data collection processes, data preprocessing, neural network models employed in this study and the result evaluation processes. Section IV deals with the result study, and the concluding remarks are presented in Section V.

## Background study

Because of the advent of deep learning and its implementation in image processing and classification, a considerable amount of research has grown in deep learning applications, specifically in autodiagnosis of radiological findings and segmentation tasks. In the classification task that employs a transfer learning technique, ResNet^14^ inception^15^, exception^16^, EfficientNet^17^ networks have grown in prominence over time. Transfer learning is an approach in deep learning where pre-trained models are used as the starting point for specified tasks. It refers to the application of a previously learnt model to a new challenge. In recent days, popularly used transformer models for natural language processing are being introduced in computer vision tasks, which are showing supremacy and good results over other models while doing classification tasks. The Vision transformer (ViT)^18^ and several variations of the Vision transformer, like the Big Transformer (BiT)^19^, EANet (External Attention Transformer)^20^, Compact Convolutional Transformer (CCT)^21^, and Swin Transformer (Shifted Window Transformer)^22^ are utilizing attention based mechanism where basic analysis unit is pixels of images.

Numerous deep learning methods are employed in research on kidney disease classification. The renal ultrasound pictures are enhanced with a median filter, a Gaussian filter, and morphological operations in the article^23^, and then characteristics from the images are retrieved with Principal Component analysis (PCA) and the K-nearest neighbor (KNN) classifier. The authors in^24^ evaluated different traditional ML algorithms, such as Decision Trees (DT), Random Forest (RF), Support Vector Machines (SVM), Multilayer Perceptron (MLP), K-Nearest Neighbor (KNN), Naive Bayes, and deep neural networks using Convolutional Neural Network (CNN) and got the highest F1 score of 0.853. In^25^, pre-trained DNN models such as ResNet-101, ShuffleNet, and MobileNet-v2 are used to extract features from kidney ultrasound pictures, which are then classified using a SVM, with final predictions made using the majority voting technique. The authors used ultrasound images there for classification problem and got the highest accuracy of 95.58%. The residual dual-attention module (RDA module) was employed for the segmentation of renal cysts in CT images in^26^. In^27^, the authors integrated the features of using conventional and deep transfer learning techniques, and finally, features are used by the SVM Classifier to classify normal and abnormal images using US images. In^28^, two CNN models are used consecutively, where the first CNN was used to identify the urinary tract, and the second CNN to detect the presence of stone and got 95% accuracy. An automated detection of kidney stones (i.e., having/not having stone) was proposed in^29^ using coronal Computed Tomography (CT) images and a deep learning technique, yielded a detection accuracy of 96.82%. The authors used 1,799 images there in total to train and validate the model. The authors in^30^ proposed two morphology convolution layers, modified feature pyramid networks (FPNs) in the faster RCNN and combined four thresholds. They got an area under the curve (AUC) value of 0.871. The kidney cyst image detection system for abdominal CT scan images using a fully connected CNN was developed in^31^ and the authors got a true-positive rate of 84.3%.

In summary, the efforts utilizing machine learning^32^ and deep learning^33^ approaches to classify a few kidney radiological findings have provided promising results, but the majority of the tasks, we found are performed on xray or ultrasound images.A few approaches were there with CT scan images only with dual class classification. Considering the scarcity of data and the above findings of research articles, we created a database of kidney stone, cyst and tumor CT images. We implemented three deep learning techniques (VGG16, Inceptionv3 and Resnet50) to classify four classes of kidney disease and demystified the blackbox of the models to show why our model came to a certain conclusion about a class. We also implemented the latest state-of-the-art innovations in vision learning (EANet, CCT, and Swin transformer algorithms) to classify the four classes and have shown that our model has promising accuracy which can reduce the suffering of the world population through early diagnosis of diseases.

## Methodology

**Figure 1:**
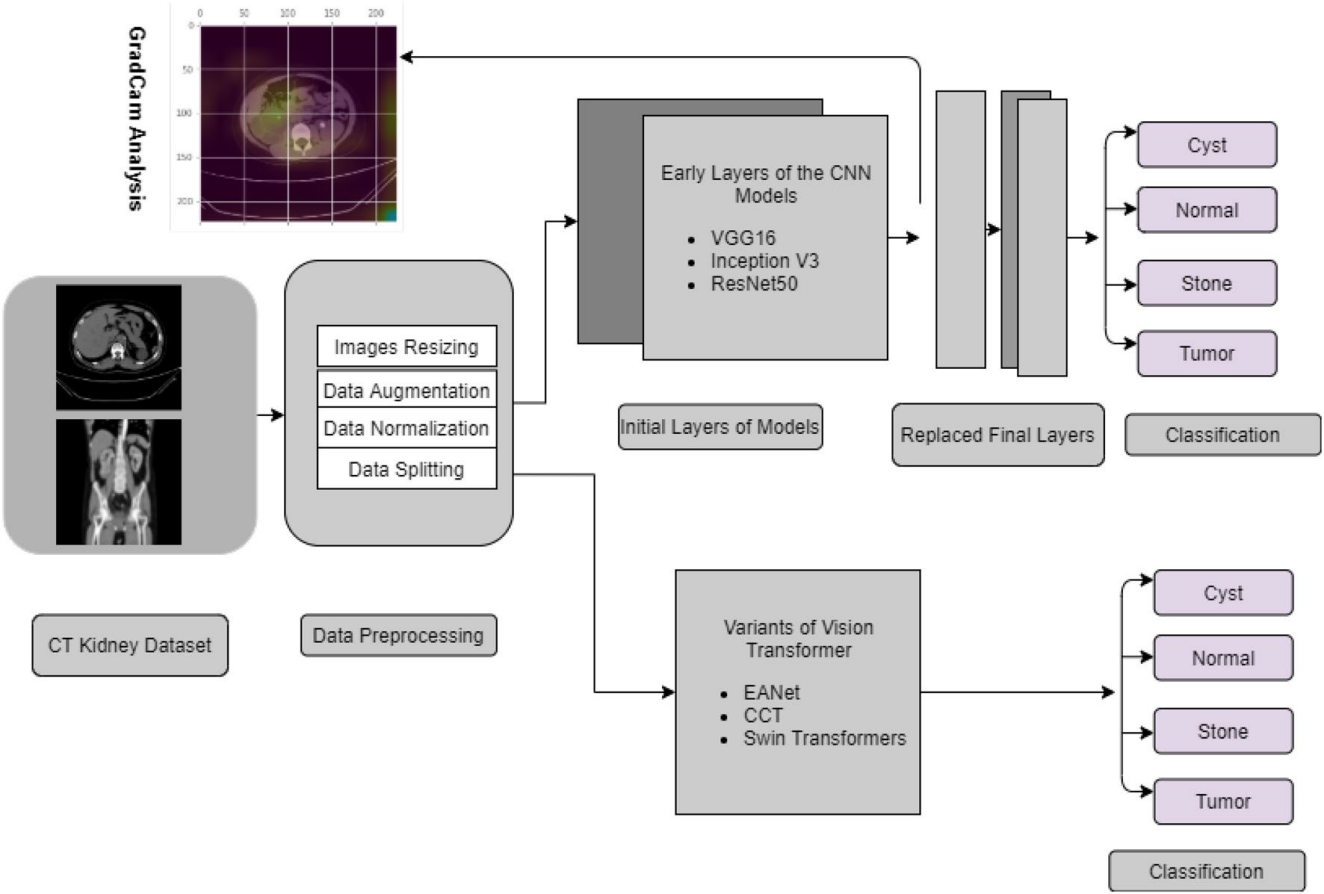
Complete Block Diagram of Experiments to diagnose Kidney tumor, cyst and stone.

We first collected and annotated the datasets to create a database for Kidney Stone, Tumor, Normal, and Cyst findings. Data augmentation, image scaling and normalization, and data splitting are among the preprocessing techniques utilized. After that, we employed six models to investigate our data, including three Visual Transformer variants (EANet, CCT, and Swin Transformer), Inception v3, and Vgg16 and Resnet 50. The model’s performance was evaluated using previously unseen data. The Block contains details about our experiment’s diagram can be found in Fig. 1

The methodology is presented in this part in the following order: dataset description, image preprocessing, neural network models, and evaluation strategies of the experiments.

### DataSet description

The dataset was collected from PACS (Picture archiving and communication system) and workstations from a hospital in Dhaka, Bangladesh where patients were already diagnosed with having a kidney tumor, cyst, normal or stone findings. All subjects in the dataset volunteered to take part in the research experiments, and informed consents were obtained from them prior to data collection. The experiments and data collection were pre-approved by the relevant hospital authorities of Dhaka Central International Medical College and Hospital (DCIMCH). Besides, the data collection and experiments were carried out in accordance with the applicable rules and regulations.

Both the Coronal and Axial cuts were selected from both contrast and non-contrast studies with protocol for the whole abdomen and urogram. The Dicom study was then carefully selected, one diagnosis at a time, and from those we created a batch of Dicom images of the region of interest for each radiological finding. Following that, we excluded each patient’s information and meta data from the Dicom images and converted the Dicom images to a lossless joint photographic expert group (jpeg/jpg) image format. The Philips IntelliSpace Portal 9.0^34^ application is used for data annotation, which is an advanced image visualization tool for radiology images, and the Sante Dicom editor tool^35^ is used for data conversion to jpg images, which is primarily used as a Dicom viewer with advanced features to assist radiologists in diagnosing specific disease findings. After the conversion and annotation of the data manually, each image finding was again verified by a doctor and a medical technologist to reconfirm the correctness of the data.

**Figure 2:**
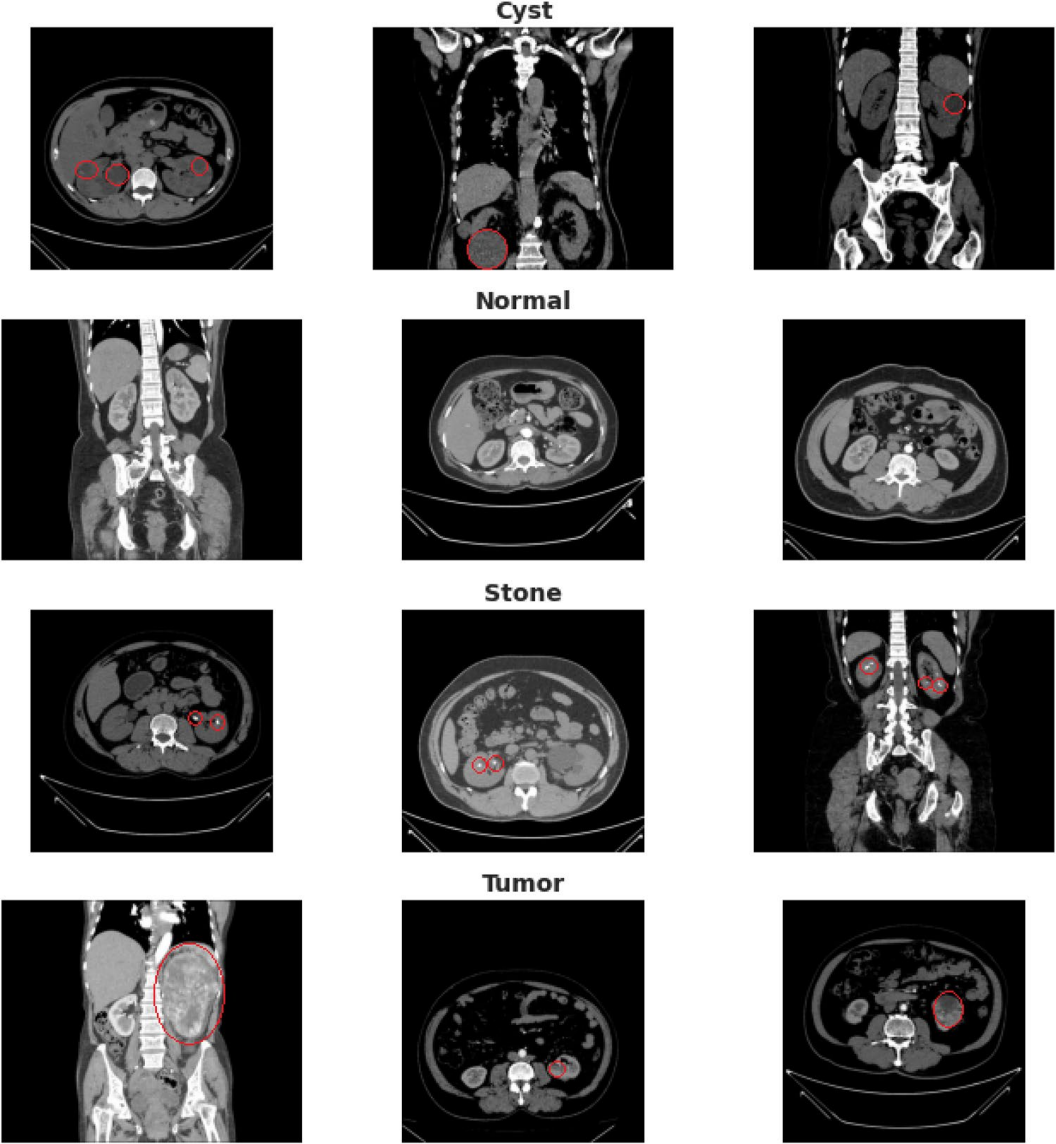
sample image data of kidney cysts, normal, stone and tumor findings.

Our created dataset contains 12,446 unique data within it in which the cyst contains 3,709, normal 5,077, stone 1,377, and tumor 2,283. The dataset was uploaded to Kaggle and made publicly available so that other researchers could reproduce the result and further analyze it. Figure 2 depicts a sample selection of our datasets. The red marks represent the finding area or region of interest that a radiologist uses to reach a conclusion for specific diagnosis classes.

**Figure 3:**
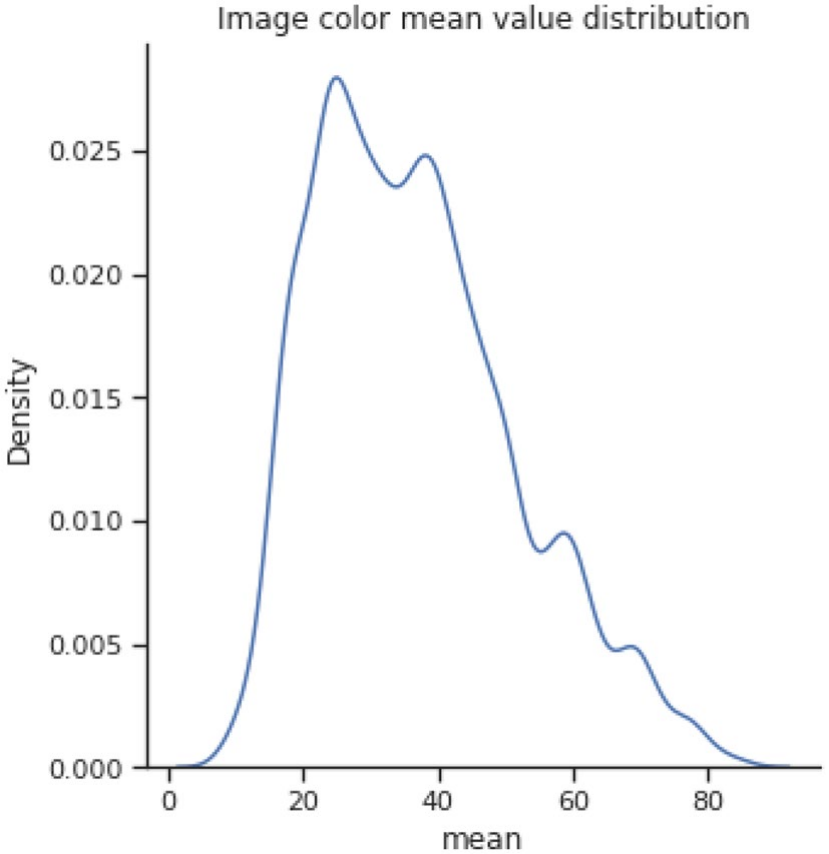
colour mean value distribution of images.

**Figure 4:**
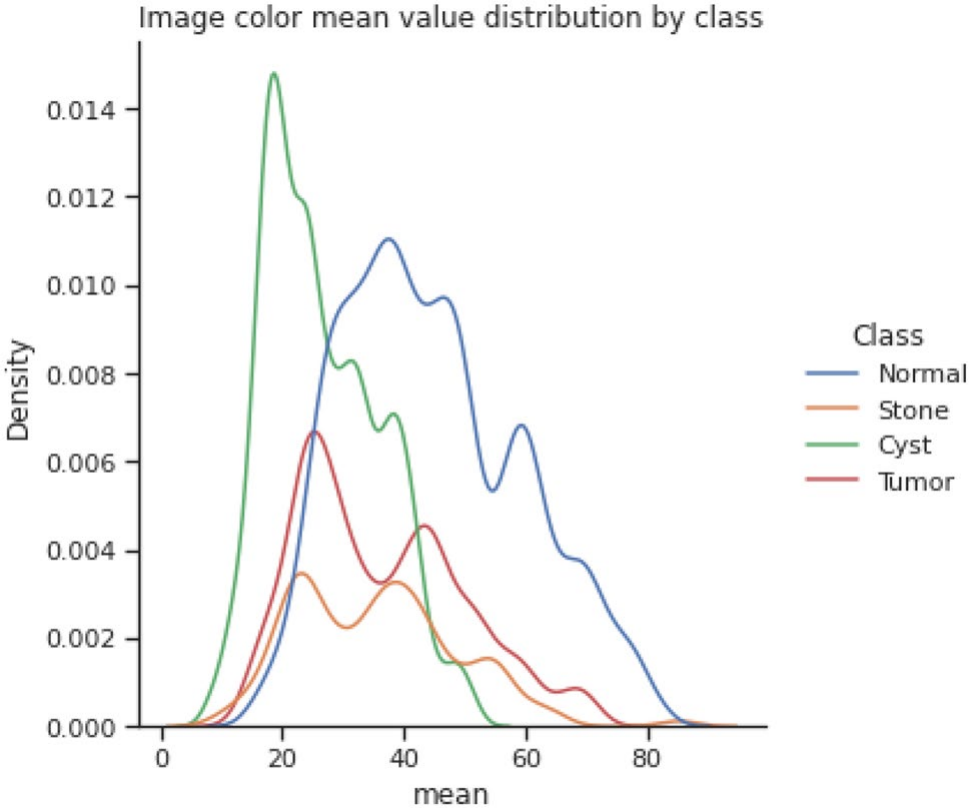
Image colour mean value distribution by class.

**Figure 5:**
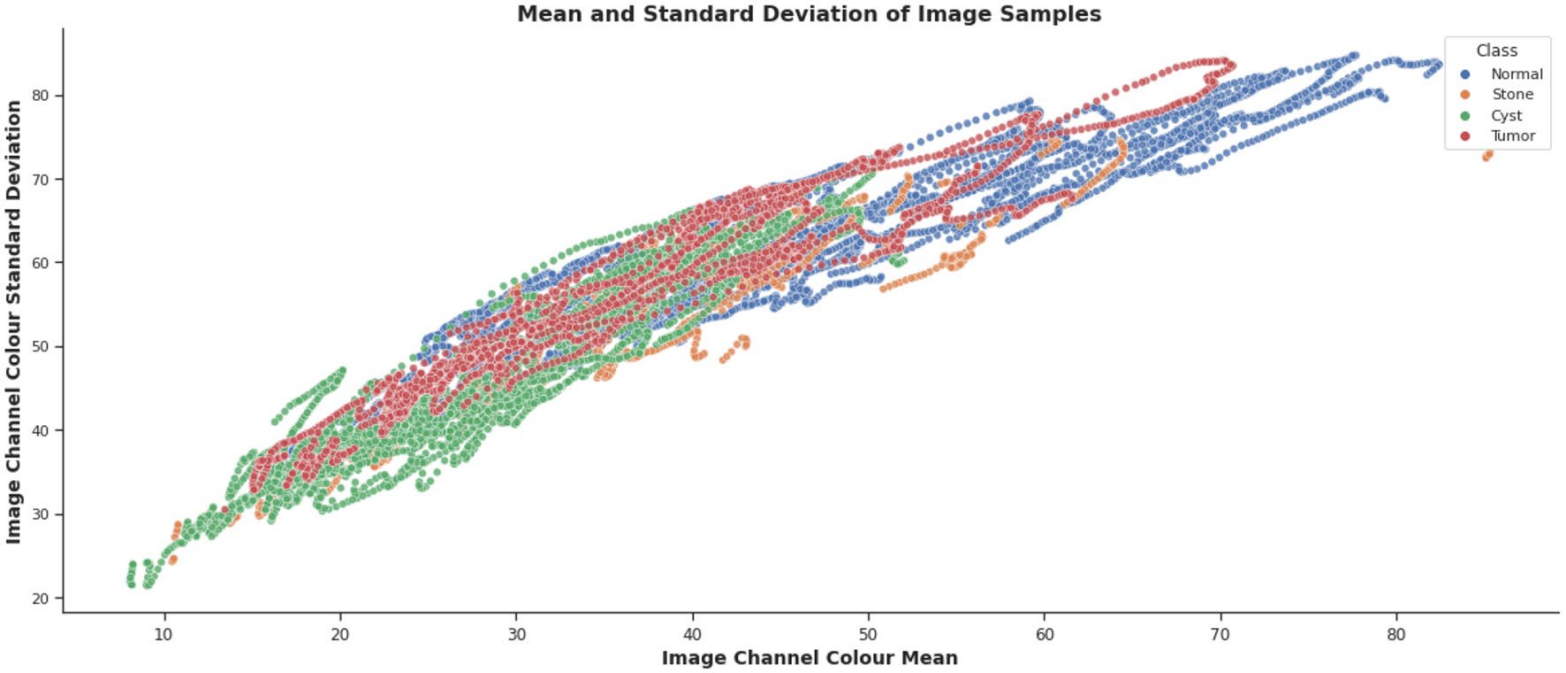
mean and standard deviation of Image samples.

Figures 3 and 4 show the image color mean value distribution and the image color mean value distribution by four classes for our dataset respectively. From both these distributions, it can be concluded that the whole dataset is very similar to the distribution of individual normal, stone, cyst, and tumor images. The mean and standard deviation of the image samples plot show that most of the images are centered, whereas stones and cysts have lower mean and standard deviation which can be visualized in Fig. 5. Since the data distributions of different renal disease classes are partially overlapped therefore, classification of cyst, tumor, and stone is not possible using only analyzing the statistical features.

### Image Processing

After converting DICOM images into jpg images, we scaled the images as per the standard size requirement of neural network models. For all the transformer variant algorithms, we resized each image to 168 by 168 pixels. Images for Inception v3 were resized to 299 by 299 pixels, while images for VGG16 and Resnet were reduced to 224 by 224 pixels.We then randomized all the images and took 1,300 examples of each diagnosis for the models’ consideration to avoid data imbalance problems, as we have 1,377 images available for the kidney stone category. The rotation operation for image augmentation was performed by rotating the images clockwise at an angle of 15 degrees. We evaluated all the models using a scheme where 80% of the images were taken to train the model and 20% to test the data. Within 80% of the training images, we took 20% to validate the model to avoid overfitting. The dataset is normalized using Z-normalization^36^ using following (1):

1 $$\begin{aligned} {\hat{X}} = \frac{X[:i]-\mu _i}{\sigma _i} \end{aligned}$$

Here, $\mu _i$ is the mean and $\sigma _i$ is the standard deviation value of the feature.

### Transfer Learning Based Neural Network Models

From the dataset, i. e., the CT KIDNEY DATASET: Normal-Cyst-Tumor and Stone, we randomly chose 1300 images of each class and trained our six models. All the neural network models were trained on Google Colab Pro Edition with 26.3 GB of GEN Ram and 16160 MB of GPU RAM using Cuda version 11.2. All the models were trained with a batch size of 16 and up to 100 epochs.

#### Vgg16

In our experiment, the 16-layer VGG 16^37^ model was tweaked in the last few layers by using the first 13 layers of the original VGG16 model, and we added average pooling, flattening, and a dense layer with a relu activation function. A dropout and finally another dense layer is added to classify the normal kidney as well as cysts, tumors, and stones. The total number of parameters in our modified VGG16 is 14,747,780, out of which 4,752,708 are the trainable parameters and 9,995,072 are the non-trainable parameters. Table 1 shows the number of parameters of the different models used in our study.

#### Resnet50

To avoid the vanishing gradient problem, and performance degradation of deep neural networks, skip connections are being used in the original Resent model. We utilized 50-layer resnet50^14^ models and modified them as the same as the Vgg16 and Inception v3 layers in the final few layers to achieve the classification task. The total number of parameters in our modified Resnet 50 model is 23,719,108. Trainable and nontrainable parameters are 135,492 and 23,583,616 respectively.

#### Inception v3

A variant of the Inception family neural network, Inception v3 based on Depthwise Separable Convolutions, is used in our study to classify images. Similar to VGG 16, we modified the original Inception v3^15^ model in the last few layers, by keeping all the layers except the last three. We added average pooling, flattening, a dense layer, a dropout, and finally a dense layer to do the classification task. The total number of parameters in inception v3 is 22,327,396 with 524,612 trainable parameters. The total number of non-trainable parameters is 21,802,784.

**Table 1:** No of parameters of different models.

Model	Total Parameter	Trainable parameter
VGG16	14,747,780	4,752,708
Inception v3	22,327,396	524,612
Resnet50	23,719,108	135,492
EANet	600,907	600,900
Swin Transformers	412,788	396,372
CCT	407,365	407,365

### Transformer Based Models

#### External Attention Transformer(EANet)

**Figure 6:**
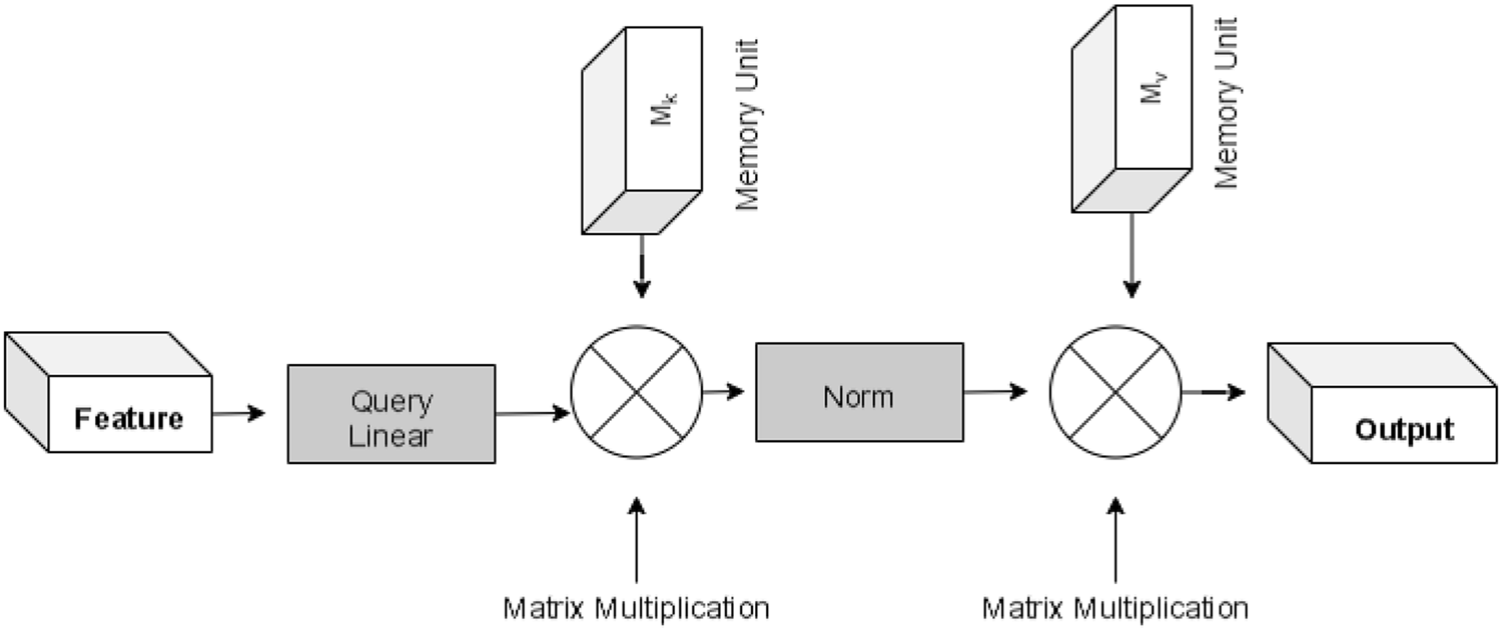
External attention of EANet model.

Though the transformer-based models were popular in Natural Language Processing, the recent advent of the vision transformer is gaining popularity over time, which utilizes the transformer architecture that uses self-attention to sequences of image patches^18^. The sequence of image patches is the input to the multiple transformer block in this case, which uses the multihead attention layer as a self-attention mechanism. A tensor of batch_size, num_patches, and projection_dim is produced by transformer blocks, which may subsequently be passed to the classifier head using softmax to generate class probabilities. One variant of the Vision Transformer EANet is shown in Fig. 6. EANet^20^ utilizes external attention, based on two external, small, learnable, and shared memories, $M_k$ and $M_v$. The purpose of EANet is to drop patches that contain redundant and useless information and hence improve performance and computational efficiency. External attention is implemented using two cascaded linear layers and two normalization layers. EANet computes attention between input pixels and external memory unit via following formulas (2), (3)

2 $$\begin{aligned} \mathrm {A} =Norm\left( \mathrm {F}\mathrm {M}_\mathrm {k}^\mathrm {T} \right) \end{aligned}$$

Finally, input features are updated from $M_v$ by the similarities in Attention A.

3 $$\begin{aligned} \mathrm {F}_\mathrm {out} =\mathrm {A}\mathrm {M}_\mathrm {v} \end{aligned}$$

We utilized TensorFlow Addons packages to implement EANet. After doing data augmentation with random rotation at scale 0.1, random contrast with a factor of 0.1, and random zoom with a height and width factor of 0.2, we implemented the patch extraction and encoding layer. Following that, we implemented an extraneous attention block, and transformer block. The output of the transformer block is then provided to the classifier head to produce class probabilities to calculate the probabilities of kidney normality, stone, cyst, and tumor findings.

#### Compact convolutional transformer(CCT)

**Figure 7:**
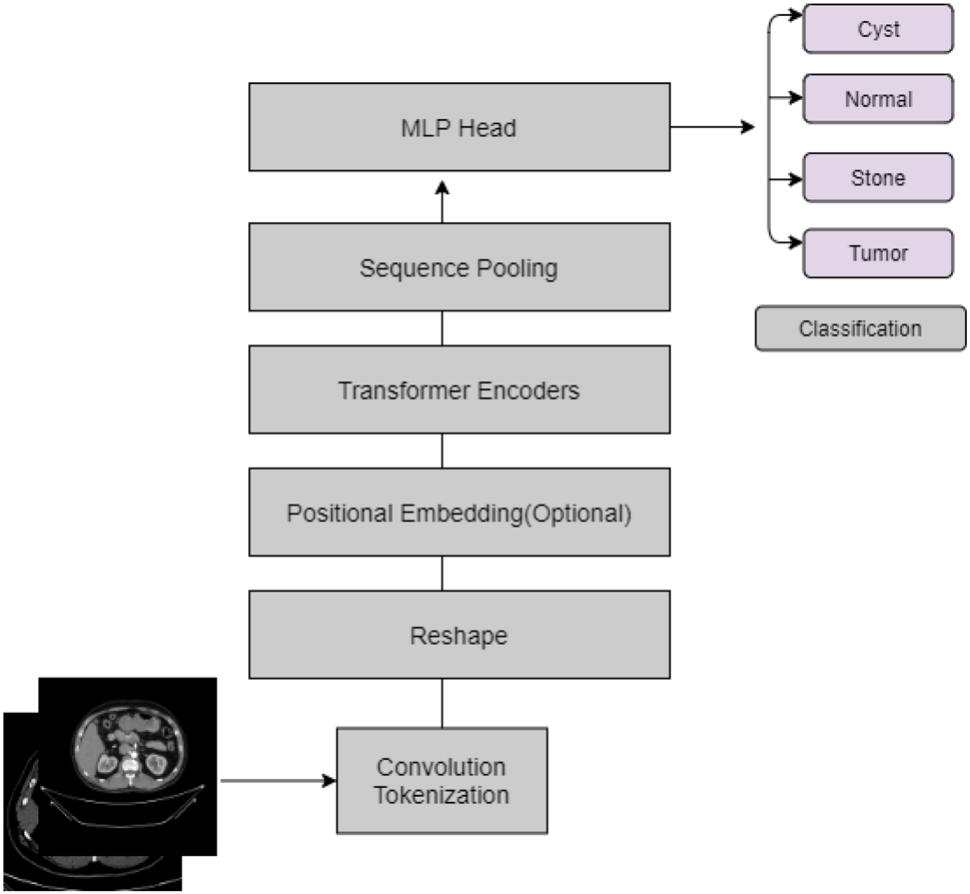
Compact Convolutional Transformer (CCT) used in the study.

Convolution and transformers are combined on CCT to maximize the benefits of convolution and transformers in vision. Instead of using non overlapping patches, which are used by the normal vision transformer in CCT^21^, the convolution technique is used where local information is well-exploited. Figure 7 illustrates the CCT procedure.

CCT is run using TensorFlow Addons, where first data is augmented using random rotation at scale 0.1, random contrast with a factor of 0.1, and random zoom with a height and width factor of 0.2.To avoid gradient vanishing problems in CCT, a stochastic depth^38^ regularization technique is used, which is very much similar to dropout except, in stochastic depth, a set of layers is randomly dropped. In CCT, In CCT, after doing convolution tokenization, data is fed to a transformer encoder and then sequence pooling. Following the sequence pooling MLP head gives the probabilities of different classes of the kidney diagnosis. The total number of parameters in our proposed CCT model has 407,365 parameters and all the parameters are trainable.

**Figure 8:**
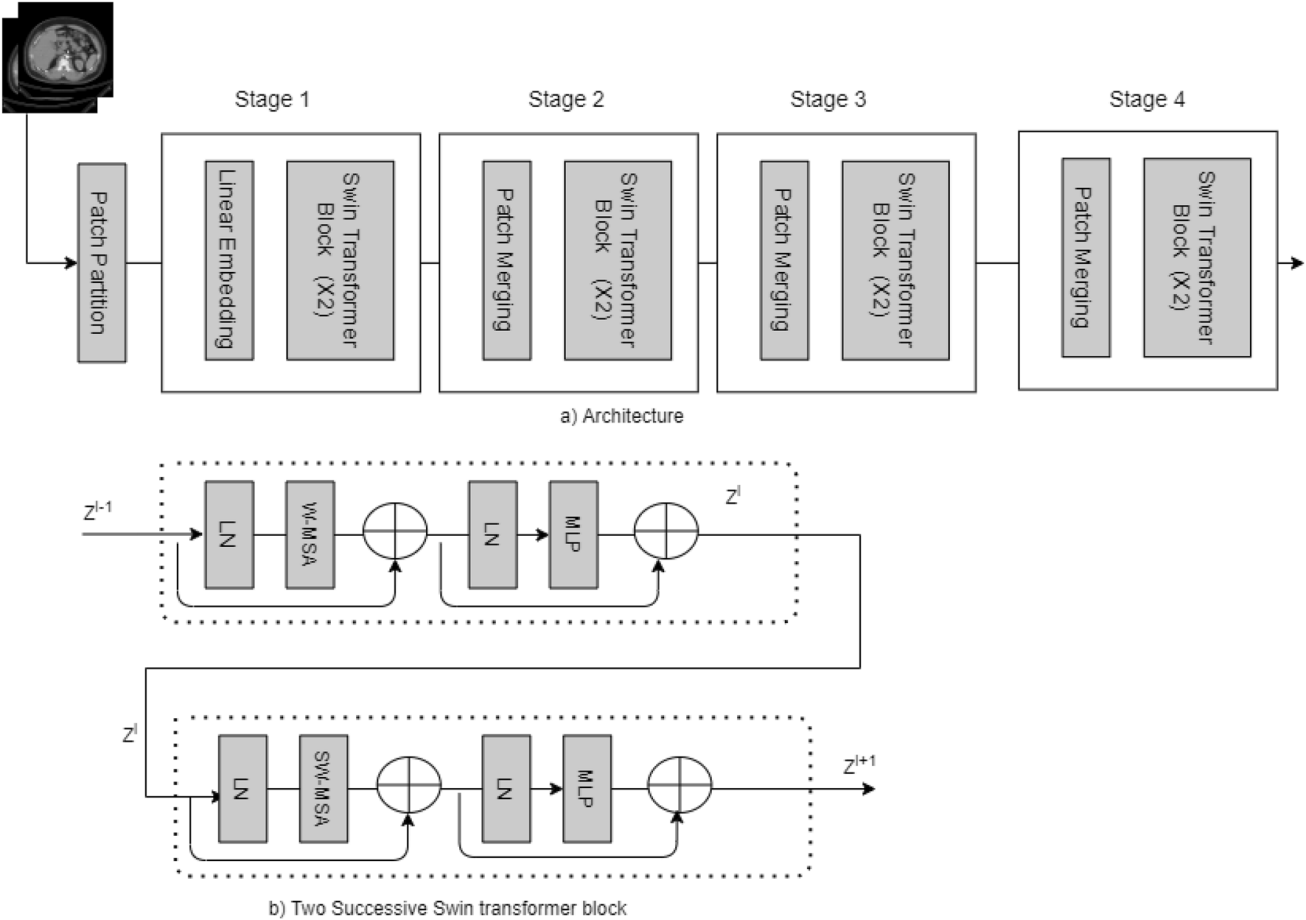
Shifted Window Transformer(Swin Transformer) diagram used in the study.

#### Shifted Window Transformers (Swin Transformers)

Another variant of the Vision Transformer is the Swin Transformer^22^, which is another powerful tool in computer vision. Detailed block diagram of the Swin transformer is shown in Fig. 8. In the picture, we can see four unique building blocks. First, the input image is split into patches by the patch partition layer. The patch is then passed to the linear embedding layer and the swin transformer block. The main architecture is divided into four stages, each of which contains a linear embedding layer and a swin transformer block multiple times. The Swin transformer is built on a modified self-attention and a block that includes multi-head self-attention (MSA), layer normalization (LN), and a 2-Layer Multi-Layer perceptron (MLP). In this paper, we utilized the swin transformer to tackle the classification problem and diagnose kidney cysts, tumors, stones, and normal findings.

### Performance Evaluation Methods

The quantitative evaluation of all the six models is calculated based on the parameters of accuracy, sensitivity or recall, precision, or PPV. True positive(*TP*), false positive(*FP*), true negative(*TN*), and false negative(*FN*) samples are used to calculate the accuracy (4), precision (5), sensitivity (6) . The recall, also known as sensitivity, is the model’s ability to identify all relevant cases within a data set. The number of true positives is divided by the number of true positives plus the number of false negatives. It refers to the study’s capability to appropriately identify sick patients with the disease. Diseases are frequently defined as a positive category in medical diagnosis. Omitting this (positive category) has serious consequences, such as misdiagnosis, which can lead to patient treatment delays. As a result, high sensitivity or recall is critical in medical image diagnosis. Precision (PPV) is necessary when out of all the examples that are predicted as positive, if we desire to know how many are really positive. With precision, the number of true positives is divided by the number of true positives plus the number of false positives. High precision is desired in the medical imaging domain. The F1 score (7) of all the models is calculated by using those models’ sensitivity and precision. The following formulas are applied to accuracy, precision, sensitivity, and F1 score.

4 $$\begin{aligned}&\mathrm {Accuracy}_\mathrm {i} =\frac{\mathrm {TP}_\mathrm {i} +\mathrm {TN}_\mathrm {i}}{\mathrm {TP}_\mathrm {i} +\mathrm {TN}_\mathrm {i}+\mathrm {FP}_\mathrm {i} +\mathrm {FN}_\mathrm {i}} \times 100 \% \end{aligned}$$

5 $$\begin{aligned}&\mathrm {Precision}_\mathrm {i} =\frac{\mathrm {TP}_\mathrm {i}}{\mathrm {TP}_\mathrm {i} +\mathrm {FP}_\mathrm {i}} \end{aligned}$$

6 $$\begin{aligned} \mathrm {Sensitivity}_\mathrm {i} =\frac{\mathrm {TP}_\mathrm {i}}{\mathrm {TP}_\mathrm {i} +\mathrm {FN}_\mathrm {i}} \end{aligned}$$

7 $$\begin{aligned}&\mathrm {F1\_score}_\mathrm {i} =2\times \frac{\mathrm {Precision}_\mathrm {i} \times \mathrm {Sensitivity}_\mathrm {i}}{\mathrm {Precision}_\mathrm {i} +\mathrm {Sensitivity}_\mathrm {i}} \end{aligned}$$

Where,

- i=Kidney Tumor or Cyst or Normal or Stone class for the classification task.
- TP= True Positive
- FN= False Negative.
- TN=True Negative

Furthermore, we plotted a receiver operating characteristic (ROC) curve with the transverse axis being the false positive rate (FPR) and the longitudinal axis being the true positive rate (TPR). The AUC, or area under the ROC curve, measures the ROC curve’s ability to classify inputs. The higher the AUC, the better the classification capabilities of the model. The area under the curve is also calculated for each developed model, and finally, all the models are compared to take a decision on which model is superior compared to other models.

**Figure 9:**
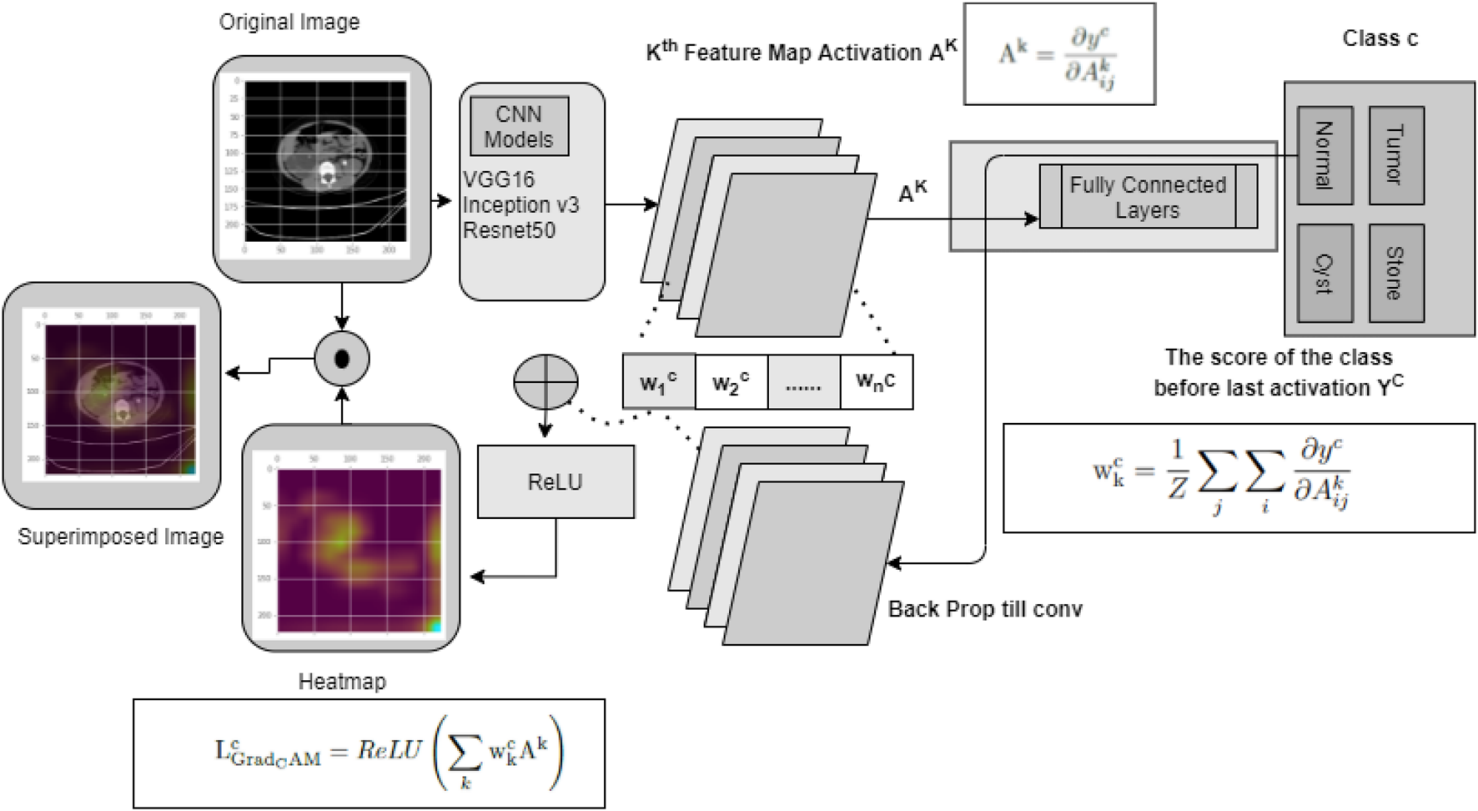
The complete process for Gradcam analysis for Kidney stone, cyst, tumor and normal classes.

This paper used the gradient weighted Class Activation Mapping (GradCAM)^39^ algorithm to make models more transparent by visualizing the input areas crucial for model predictions in the last convolution layers of CNN networks. Figure 9 describes complete process for Gradcam analysis in our paper.

First, we passed a picture through the model to get a prediction, and then we developed the image’s class prediction based on the prediction value. After that, we computed the gradient of the class known as Feature Map activation $\hbox {A}^k$(8).

8 $$\begin{aligned} \mathrm {A}^\mathrm {k} = \frac{\partial y^c}{\partial A^k_{ij}} \end{aligned}$$

These gradients flowing back are global-average-pooled across the width and height dimensions (indexed by i and j, respectively) to calculate neuron significance weights (9).

9 $$\begin{aligned} \mathrm {w}_\mathrm {k}^\mathrm {c} =\frac{1}{Z}\sum _{j} \sum _{i}\frac{\partial y^c}{\partial A^k_{ij}} \end{aligned}$$

Then neuron significance weights and feature map activations are summed and applied the Relu activation to the summed result to get the GradCam(10).

10 $$\begin{aligned} \mathrm {L}_\mathrm {Grad_CAM}^\mathrm {c} =ReLU\left( \sum _k\mathrm {w}_\mathrm {k}^\mathrm {c} \mathrm {A}^\mathrm {k}\right) \end{aligned}$$

Where,

- $\hbox {A}^k$= feature map activation
- ${w}_\mathrm {k}^\mathrm {c}$= neuron significance weights

We created a visualization by superimposing the original image with the heatmap. This visualization helps us to determine why our model came to the conclusion that an image may belong to a certain class, like kidney tumor, cyst, normal, or stone.

## Result analysis

**Figure 10:**
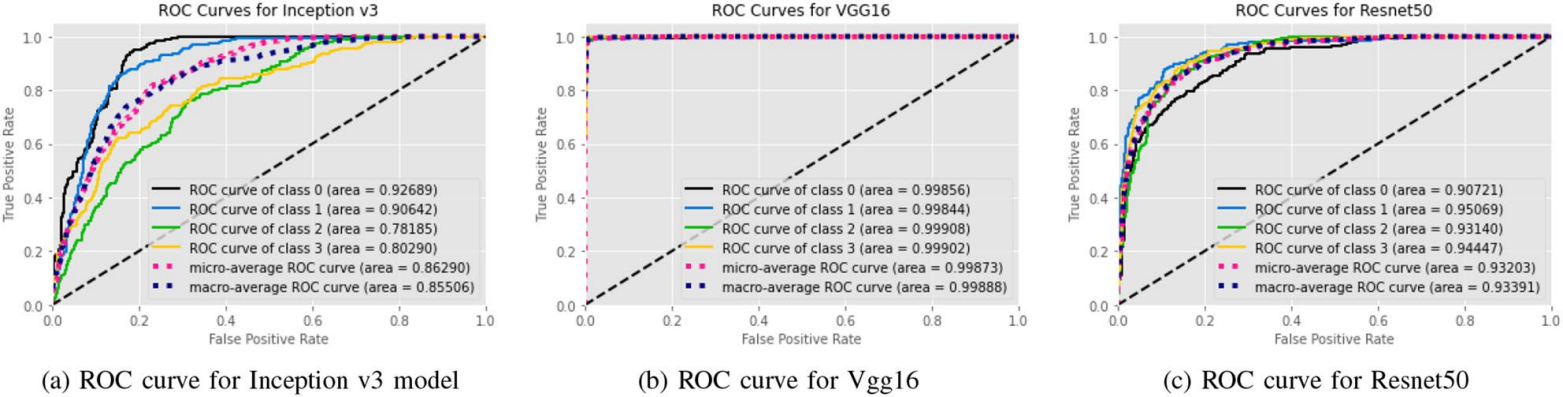
ROC curves for Transfer Based Models Used in Our study.

The results of the implemented six models using different tests are evaluated by calculating the accuracy, recall, F1 score (F1), accuracy (Acc), positive predictive value (PPV), and ROC curve area of interest (AUC) from unseen data. We used Tenfold cross-validation and the result was averaged to produce the ROC curve, confusion matrix, and evaluation matrices. Table 2, Figs. 10 and 12 summarizes the performance of the six networks studied in this paper. Figure 14 presents us with the gradcam analysis of the Inception v3, Resnet50, and Vgg16 models. Figure 12 provides the ROC curves for Transfer and Transformer based models consecutively. Figures 10 and 12 shows the normalized Confusion Matrices for Transfer and Transformer based models consecutively.

**Table 2:** MEASURES OF PERFORMANCE FOR THE SIX MODELS STUDIED IN THE RESEARCH.

Models	Accuracy	Class	Precision (PPV)	Recall (Sensitivity)	F1 Score	AUC
EANet	77.02%	Cyst	0.593	1	0.745	0.98
		Normal	0.896	0.848	0.871	0.98
		Stone	0.845	0.495	0.624	0.91
		Tumor	0.93	0.777	0.847	0.97
Swin Transformers	99.30%	Cyst	0.996	0.996	0.996	0.99993
		Normal	0.996	0.981	0.988	0.9998
		Stone	0.981	0.989	0.985	0.99975
		Tumor	0.993	1	0.996	1
CCT	96.54%	Cyst	0.968	0.923	0.945	0.99605
		Normal	0.989	0.975	0.982	0.99841
		Stone	0.94	1	0.969	0.99924
		Tumor	0.964	0.964	0.964	0.99723
VGG16	98.20%	Cyst	0.996	0.968	0.982	0.99856
		Normal	0.985	0.973	0.979	0.99844
		Stone	0.966	0.988	0.977	0.99908
		Tumor	0.982	0.996	0.989	0.99902
Inception v3	61.60%	Cyst	0.645	0.826	0.724	0.92689
		Normal	0.584	0.898	0.708	0.90642
		Stone	0.568	0.462	0.509	0.78185
		Tumor	0.76	0.295	0.425	0.8029
Resnet50	73.80%	Cyst	0.735	0.641	0.685	0.90721
		Normal	0.77	0.79	0.78	0.95069
		Stone	0.745	0.692	0.717	0.9314
		Tumor	0.706	0.827	0.762	0.94447

**Figure 11:**
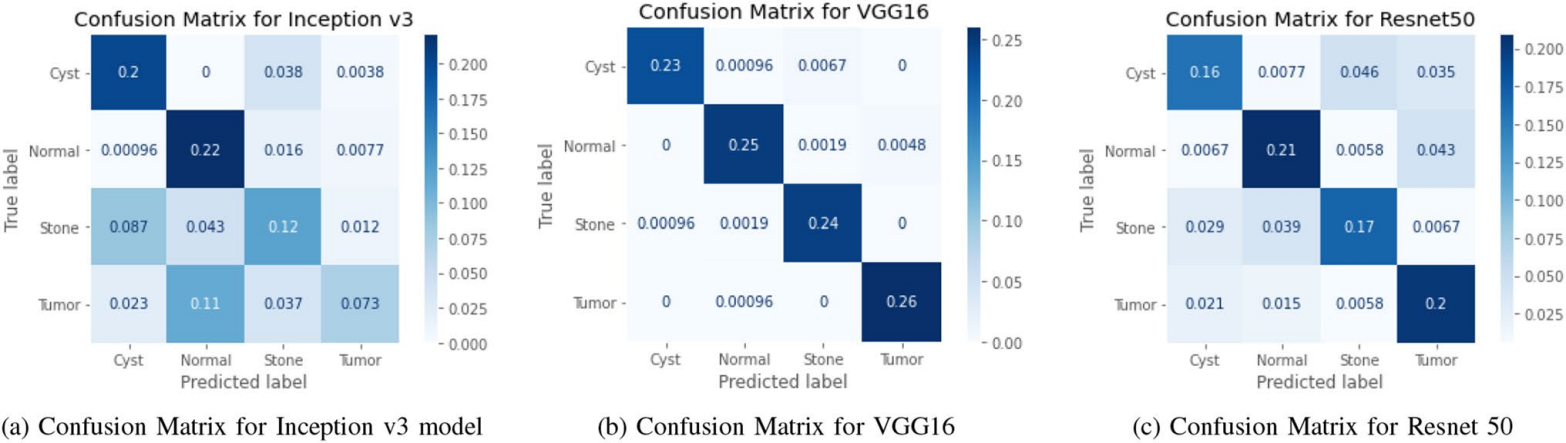
Confusion Matrices for Transfer Based Models Used in Our study.

**Figure 12:**
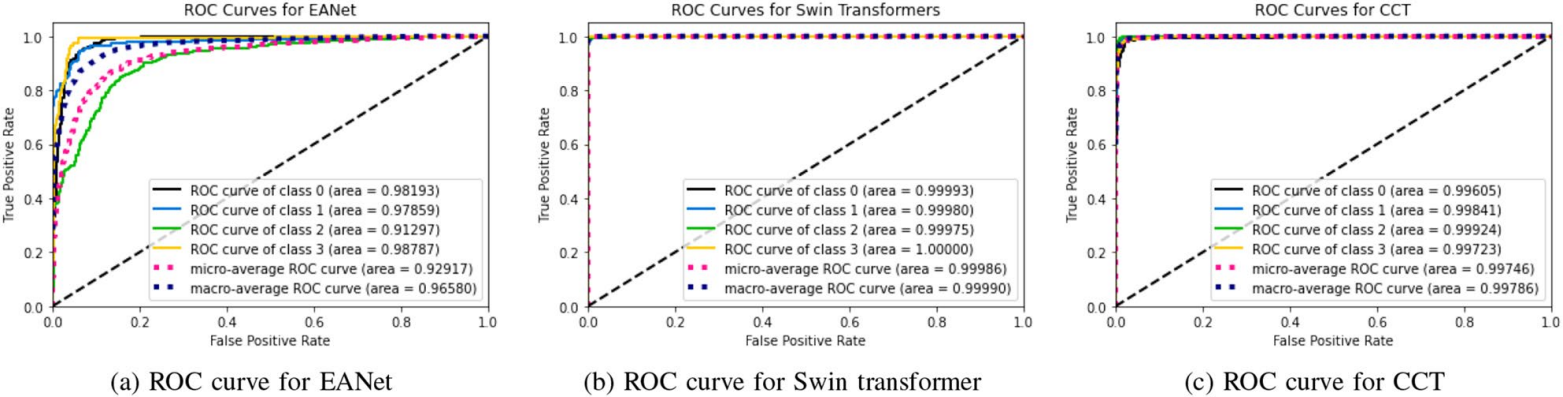
ROC curves for Transformer Based Models Used in Our study.

**Figure 13:**
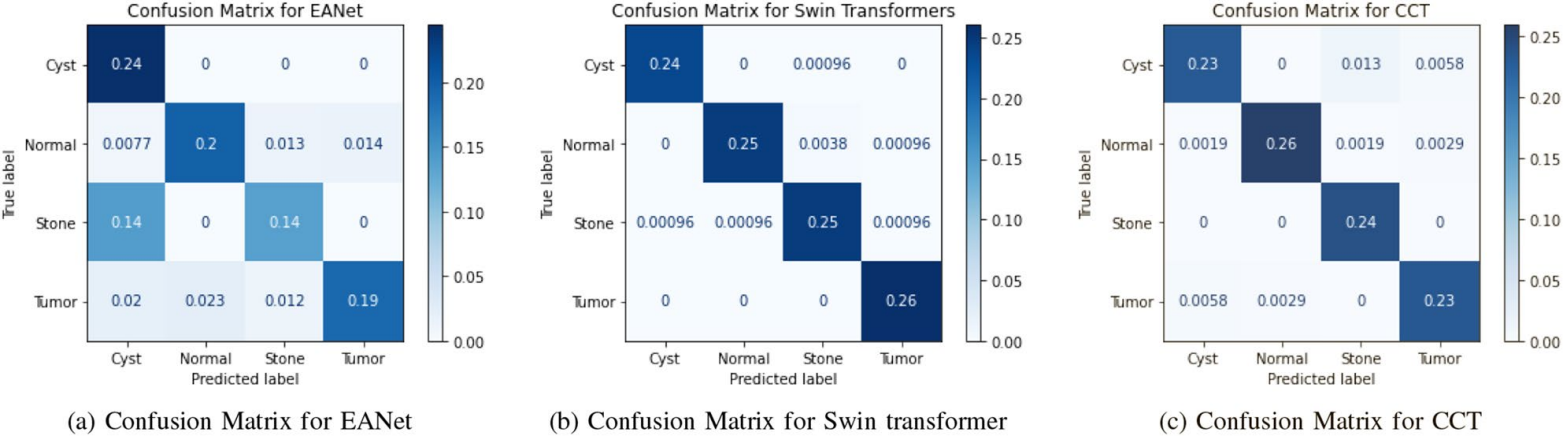
Confusion Matrices for Transformer Based Models Used in Our study.

**Figure 14:**
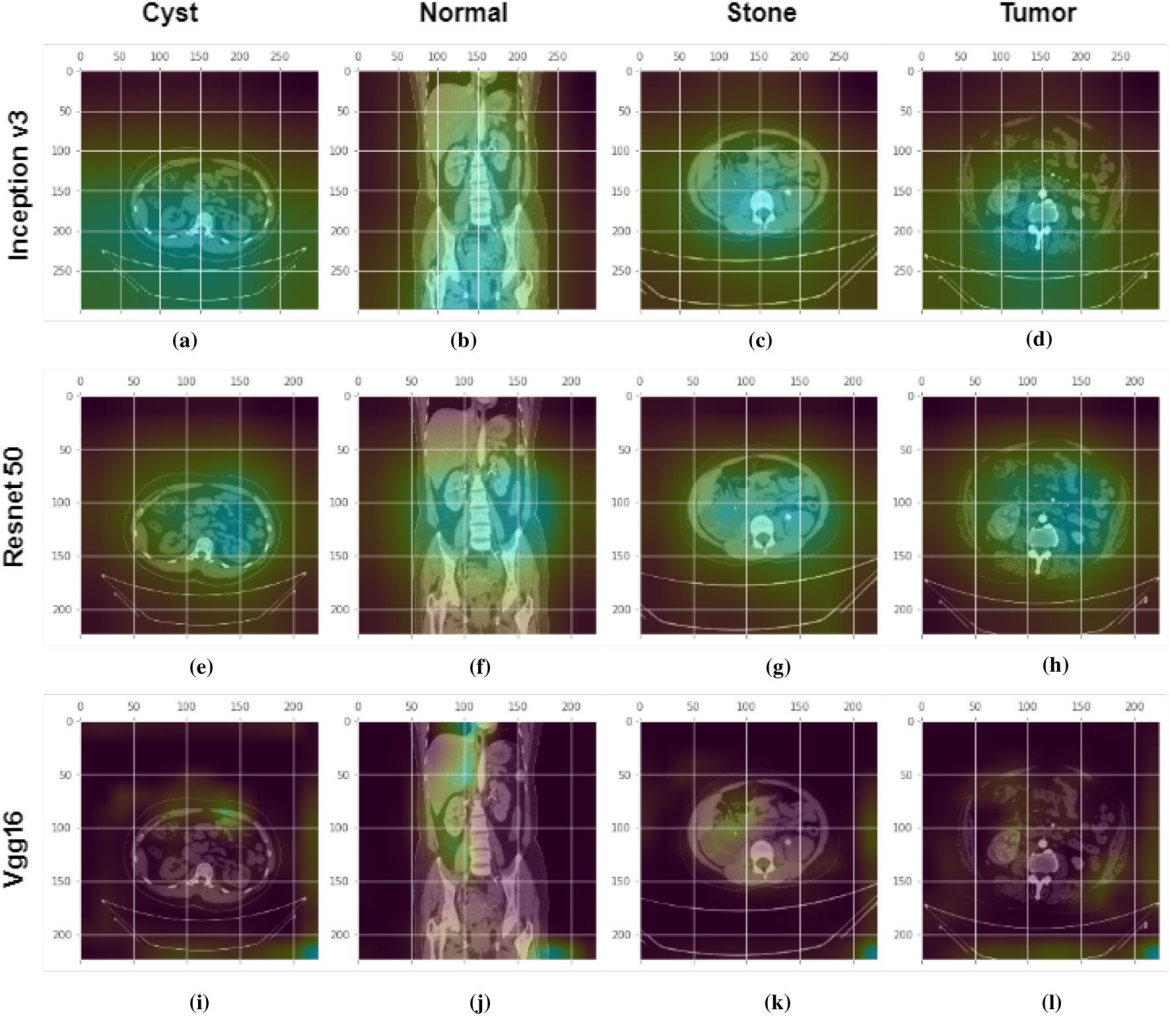
GradCam analysis of kidney Cyst, Normal, Stone and Tumor class photos at the final convolution layer in the Inception v3, Vgg16, and Resnet models. First row: shows the Gradcam images from inception v3 model for different classes. Second row: shows the Gradcam images from Resnet50 model for different classes. Third row: shows the Gradcam images from Vgg16 model for different classes. The GradCam activation mapping for the xray image is shown in the second row. The first, second, third, and fourth columns are for kidney cysts, normal, stone, and tumor classes respectively.

From the table 2, we can see that the InceptionV3 model performed worse with our dataset and gave an accuracy of 61.60%. EANet and Resnet 50 performed moderately by giving accuracy of 77.02% and 73.80%. CCT, VGG16 and Swin Transformers provided accuracy of 96.54%, 98.20% and 99.30% accuracy respectively. The Swin transformer, which is a transformer-based model, is outperforming all the other models in respect of accuracy.

The Swin Transformer is providing reasonable recall while detecting cyst, normal, stone, and tumor class images and providing a recall of 0.996, 0.981, 0.989, and 1 consecutively. Higher recall means there is the lowest chance of misdiagnosing the cyst, normal, stone, and tumor class images. From the table we can see, the Swin transformer is providing a recall of 1 for kidney stone classes and it is good at detecting kidney tumor classes, whereas CCT is good at detecting stone class images and providing a recall of 1 for the stone class images. However, for the other class images, recall for the CCT model is slightly lower than the Swin transformer model and provides a recall of 0.923, 0.975, and 0.964 for the cyst, normal, and tumor class images, respectively.

From the transfer learning based approaches, VGG16 provides a recall of 0.968, 0.973, 0.988, and 0.996 respectively for Kidney Cyst, Normal, Stone, and Tumor class images. But Inception v3 and Resnet are providing lower recall for all the classes. The recall for the Kidney Tumor class is 0.295 for the Inception v3 model and 0.462 for the Kidney Stone classes. This means the Resnet model in our study is the least effective at detecting kidney tumors and kidney stones. Since in medical image diagnosis recall is a priority matrix to consider, a model built based on Resnet and Inception v3 can’t be used in diagnosis in our case.

From the transformer based model, we can see in the table 2 the precision is highest for the Swin transformer model and provides 0.996, 0.996, 0.981, and 0.993 respectively for Kidney Cyst, Normal, Stone, and Tumor class images. From the transfer based approach, we can see VGG16 is providing better precision than Inception V3 and Resnet50.

For the cyst, normal, stone, and tumor classes, the highest F1 score is provided by the swin transformer also, and the numbers are 0.996, 0.998, 0.985, and 0.996 consecutively. The Swin transformer also provides the highest precision for Stone and Tumor classes, and readings are 0.981 and 0.993. For the cyst class, the Swin transformer and VGG 16 are providing the same value of 0.996, whereas for the normal class, the Swin transformer is performing better and giving a reading of 0.996. Considering the above, the Swin transformer is superior and outperforms all the models, and can be of great use in kidney medical imaging diagnosis.

From Figs. 10, 11, 12 and 13, we can see that the Area Under the ROC Curve is superior in the case of CCT, VGG16, and SWin Transformers than Resnet50, EANet, and Inception v3. AUC is closer to 1 while diagnosing Kidney Cyst, Normal, Stone, and Tumor categories for Swin Transformers, CCT, and VGG16 models. Considering precision, recall, and F1 Score, we can conclude that though VGG16 and CCT are performing well, the Swin transformer outperformed all the models. Though CCT and VGG16 can be used while diagnosing kidney stones, cysts, and tumors, Swin Transformer can be considered the most effective option.

After randomly providing four images of different classes from the CT machine in the GradCam algorithm, we analyzed the GradCam of the last convolution layer of the Transfer-based algorithm. From the Fig. 14, First row shows images that contain cysts. We can see from the Fig. 14a, e and i that VGG16 is watching a very small region (high level features) to take a decision about cyst class images, whereas Resnet50 and Inceptionv3 are looking at more dispersed regions, hence low-level features to classify. For the stone class images Fig. 14c, g and k, we can observe that Vgg 16 is watching the region of interest perfectly. Other models are watching dispersed regions, whereas VGG16 is watching a very small region to make a decision. A similar condition applies to the tumor and normal classes as well. In our case, VGG16 is predicting all the images as correct class and watching the region of interest perfectly, whereas Resnet is predicting normal findings such as tumors and stones as normal in this case and also not watching where the model should watch to make a decision. Inception V3 is also not watching the region of interest perfectly and watching more low-level features, and in this case, it predicated the tumor class as the normal class.

## Conclusion

For this work, we collected and annotated a total of 12,446 whole abdomen and urogram CT scan images containing cysts, tumors, normal, and stone findings. Exploratory data analysis of the images was performed and showed that the images from all the classes had the same type of mean colour distribution. Furthermore, this study has developed six models and out of which, three models are based on recent state-of-the-art variants of the Vision transformers EANet, CCT, and Swin transformers, and the other three are based on popularly known deep learning models, Resnet, Vgg16, and Inception v3, which are tweaked in the last few layers. A comparison of all the models performed revealed that, while VGG16 and CCT performed well, the Swin transformer outperformed all the models in terms of accuracy, providing an accuracy of 99.30%. The F1 score, precision, and recall comparisons provide evidence that the Swin transformer is outperforming all the models.Besides, compare to all the models, the Swin transformer has taken less time to train with the same number of epochs. The study has also tried to reveal the blackbox of VGG16, Resnet50, and Inception models and found that the VGG16 model is better compare to Resnet50 and Inceptionv3 by showing the desired abnormalities in the anatomy better. We believe the superior accuracy of our model based on the Swin transformer and the VGG16-based model can both be of great use in detecting kidney tumors, cysts, and stones, and can reduce the pain and suffering of patients.
